# Case Report: Adaptive radiotherapy in the radiation salvage of prostate cancer

**DOI:** 10.3389/fonc.2022.898822

**Published:** 2022-08-15

**Authors:** Steven K. Montalvo, Boyu Meng, Mu-Han Lin, Chunjoo Park, Neil B. Desai, Raquibul Hannan, Aurelie Garant

**Affiliations:** Department of Radiation Oncology, Harold C. Simmons Cancer Center, University of Texas Southwestern, Dallas, TX, United States

**Keywords:** stereotactic, ablative, adaptive, radiation, salvage, prostate, MR-guided

## Abstract

Adaptive radiotherapy has the potential to reduce margins, improve target coverage, and decrease toxicity to organs at risk (OARs) by optimizing radiation delivery to daily anatomic changes. Salvage for locally recurrent prostate cancer after definitive radiation remains a challenging clinical scenario given the risks to normal tissue in a setting of re-irradiation. Here, we present a case series of five patients with locally recurrent prostate cancer treated with an adaptive online linear accelerator or a 3-T MR-based linear accelerator to demonstrate excellent target coverage. All patients completed the planned treatment course with acceptable acute toxicities but a short follow-up time does not inform subacute/late toxicities.

## Introduction

Salvage re-irradiation of the intact prostate is a challenging clinical scenario. Brachytherapy has been the preferred technique for salvage re-irradiation, but it requires expertise and specialized equipment and is thus not widely available. Stereotactic ablative radiotherapy (SAbR) shows promising outcomes for use in the salvage setting and is a treatment modality available to most radiation oncology centers worldwide (1). SAbR requires precise image guidance for safe delivery with strategies including onboard cone-beam CT, fiducial placement, intrafractional motion tracking, and MR-guided radiation therapy (MRgRT). Online adaptive radiotherapy (ART) can be delivered by either cone beam CT-based (CBCT—Ethos, Varian) or MR-based (Unity, Elekta and MRIdian, Viewray) linear accelerators, and promises a new wave of treatment personalization in radiation oncology by optimizing radiation delivery in real time (2–4). It is suggested that ART may improve target coverage and concomitantly reduce unintended irradiation of mobile structures such as the bladder, rectum, and bowel by optimizing dose distribution at the time of treatment (3, 5). Consequentially, ART serves as another emerging tool to improve the safety of SAbR delivery. Here, we present the benefits and challenges of these new technologies, MRgRT and ART, in treatment delivery in five patients with locally recurrent prostate cancer who underwent salvage SAbR.

## Patient 1

**Figure 1:**
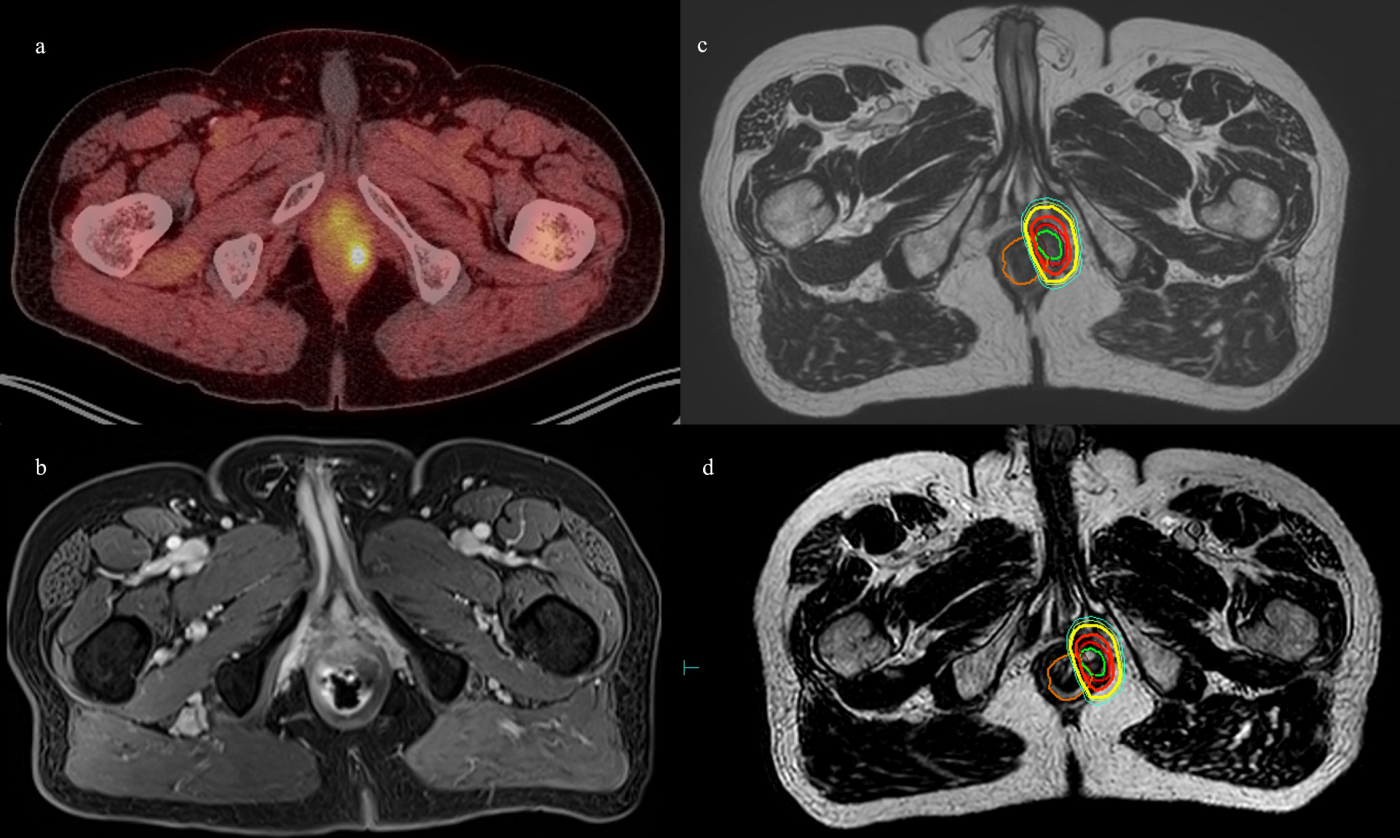
Patient 1. **(A)** Axial 18F-fluciclovine PET/CT showing left perirectal lesion. **(B)** Axial MRI showing the same. **(C)** MR simulation with planned isodose lines. **(D)** MRgRT-acquired image with delivered isodose lines. Orange = rectum, green = GTV, red = PTV, bolded red = D100%, yellow = D90%, and cyan= D80%.

Patient 1 was diagnosed at the age of 68 with high-risk localized prostate cancer: T4 with concern for pelvic sidewall involvement, a Gleason Score of 4 + 4 = 8, and an initial prostate-specific antigen (PSA) level of 61.67 ng/mL. He underwent definitive radiation involving 45 Gy in 25 fractions to the prostate and pelvic lymph nodes with a boost to 79.2 Gy in 44 fractions to the prostate and seminal vesicles, as well as 2 years of androgen deprivation with leuprolide. Upon discontinuation of androgen deprivation therapy (ADT), the patient’s PSA became detectable; however, he had no radiologic correlates for recurrence. His PSA continued to slowly rise during a period of time that he was asymptomatic. Five years after definitive radiation, the patient’s PSA rose to 2.31, and he began to experience worsening rectal pain. In the context of his elevated PSA, an F18-fluciclovine PET/CT was ordered and revealed an avid lesion in the left lateral anal canal. A follow-up MRI revealed a suspicious growing nodule measuring 1.7 × 1.4 cm that corresponded to the PET-avid lesion. The imaging findings also corresponded with the patient’s rising PSA of 2.95. He was treated in a personalized ultra-fractionated stereotactic ablative radiation (PULSAR)-based approach (6) *via* MRgRT (Unity, Elekta), originally planned for five fractions of 7 Gy spaced weekly equaling a total of 35 Gy (**Figure 1**). However, his third fraction was split into two 3.5-Gy fractions spaced a day apart due to machine quality assurance intolerance. Patient 1 ultimately received a total of 35 Gy in six fractions. The patient setup with rectum, bladder, and bowel positioning was adequately accurate so that after real-time evaluation by the treating radiation oncologist, it was decided to treat the patient with an “adapt to position” approach for each of the six fractions. This is in contrast to an “adapt to shape” approach where adaptive planning at the time of delivery is used. Patient 1 did not experience any acute toxicity during treatment. In the most recent follow-up, his PSA decreased to 1.66, 4 months post-PULSAR. He did not receive concurrent or adjuvant systemic therapy.

## Patient 2

**Figure 2:**
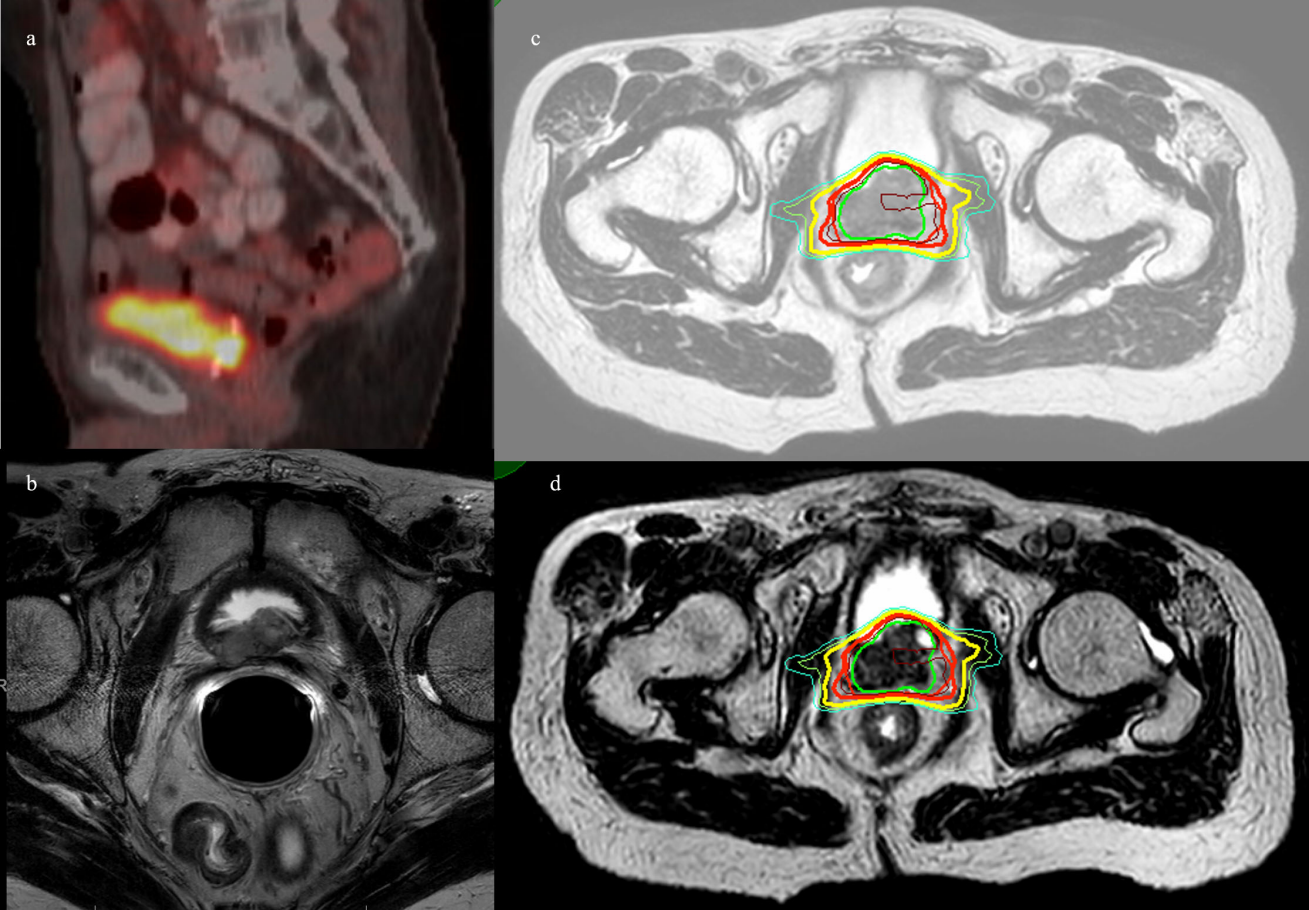
Patient 2. **(A)** Sagittal 68Ga-PSMA-11 PET/CT showing bright posterior bladder wall uptake. **(B)** Axial MRI showing posterior bladder wall lesion. **(C)** MR simulation with planned isodose lines. **(D)** MRgRT-acquired image with delivered isodose lines. Green = GTV, red = PTV, brown = D110%, bold red = D100%, yellow = D90%, and cyan = D80%.

Patient 2 was diagnosed at the age of 51 with prostate cancer and treated with a robotic-assisted laparoscopic prostatectomy. This patient showed persistent post-operative PSA and was treated with prostatic fossa radiation at the age of 56. Records for the initial prostate cancer staging and radiation therapy were unavailable, as they were performed 22 years prior to presentation. Patient 2’s PSA stabilized but never became undetectable. At 71 years of age, patient 2’s PSA increased, and at this time, leuprolide was started. An 18F-fluciclovine PET showed an avid mass in the prostatectomy bed inseparable from the left inferior bladder wall. Darolutamide was started when the patient was 73 years old due to rising PSA, suggesting castration resistance with associated posterior bladder thickening on the ultrasound. 18F-fluciclovine PET and prostate MRI at the age of 75 showed an enlarging 18F-fluciclovine-avid posterior bladder mass. Biopsy of the mass showed a Gleason Score of 4 + 5 = 9 prostate adenocarcinoma. A 68Ga-prostatic specific membrane antigen (PSMA) PET showed uptake in the prostatectomy bed, but no other areas of focal uptake. A cystoscopy did not show an intraluminal lesion or stricture. After a multi-disciplinary discussion with urologists, medical oncologists, and radiation oncologists, re-irradiation of the prostatic fossa recurrent mass with SAbR was recommended given castration resistance, time from prior radiation therapy, and concern for morbidity of further progression in the prostatic fossa mass. The patient was treated with MRgRT, with 35 Gy in five fractions delivered twice per week (**Figure 2**). No acute toxicity was experienced during treatment or at the 2-month follow-up. He continued his ADT/darolutamide and his most recent post-SAbR PSA 5 months after therapy decreased to 0.09.

## Patient 3

**Figure 3:**
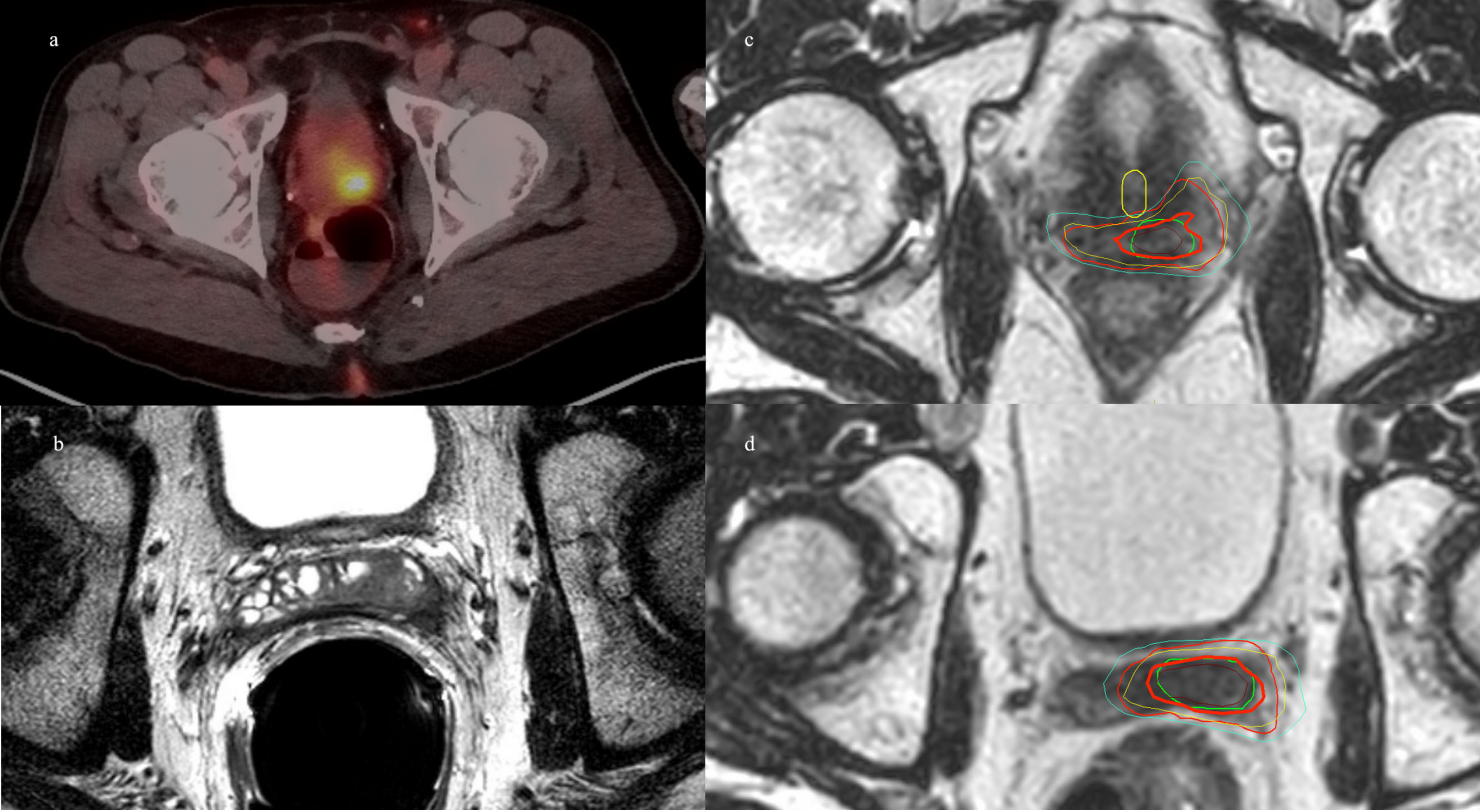
Patient 3. **(A)** Axial 18F-fluciclovine PET/CT showing bright uptake in the left posterior prostate. **(B)** MRI prostate showing left seminal vesicle invasion. **(C)** MRgRT-acquired treatment image at the level of mid-prostate. **(D)** MRgRT-acquired image at the level of seminal vesicle. Green = PTV 4,000 cGy, red = PTV 3,400 cGy, bold yellow = urethra, brown = D110%, bold red = D100%, yellow = D90%, and cyan = D80%.

Patient 3 was diagnosed at the age of 53 with low-risk prostate cancer: T1cN0, Gleason score 3 + 3 = 6, and an initial PSA of 9.26. He underwent conventionally fractionated external beam radiation of the prostate and seminal vesicles, 79.2 Gy in 44 fraction, and PSA nadir was 0.64. He met the Phoenix criteria at age 60, with PSA 2.75 prompting 18F-fluciclovine PET, which showed uptake in the left mid/base of the prostate. MRI of the prostate showed a 22 cc gland with a PIRADS 5 lesion on the left crossing midline with evidence of seminal vesicle invasion and extraprostatic extension. Template and lesion-directed MRI-guided biopsies showed Gleason score 4 + 5 = 9 prostate cancer in 4/14 cores with a total of 7/14 cores positive for > Gleason score 7 prostate cancer. He was not deemed to be a surgical candidate, and thus, he started leuprolide and bicalutamide 2 months after biopsy and, after medical oncology evaluation, started abiraterone and prednisone 3 months after biopsy with subsequent castrate testosterone and undetectable PSA. He chose to undergo MRgRT SAbR 5 months after biopsy; he was treated with RT directed at left SV and left mid-base crossing midline to 34 Gy in five fractions with SIB of gross disease to 40 Gy in five fractions (**Figure 3**). He reported no acute toxicity and continued to have an undetectable PSA post-SAbR.

## Patient 4

**Figure 4:**
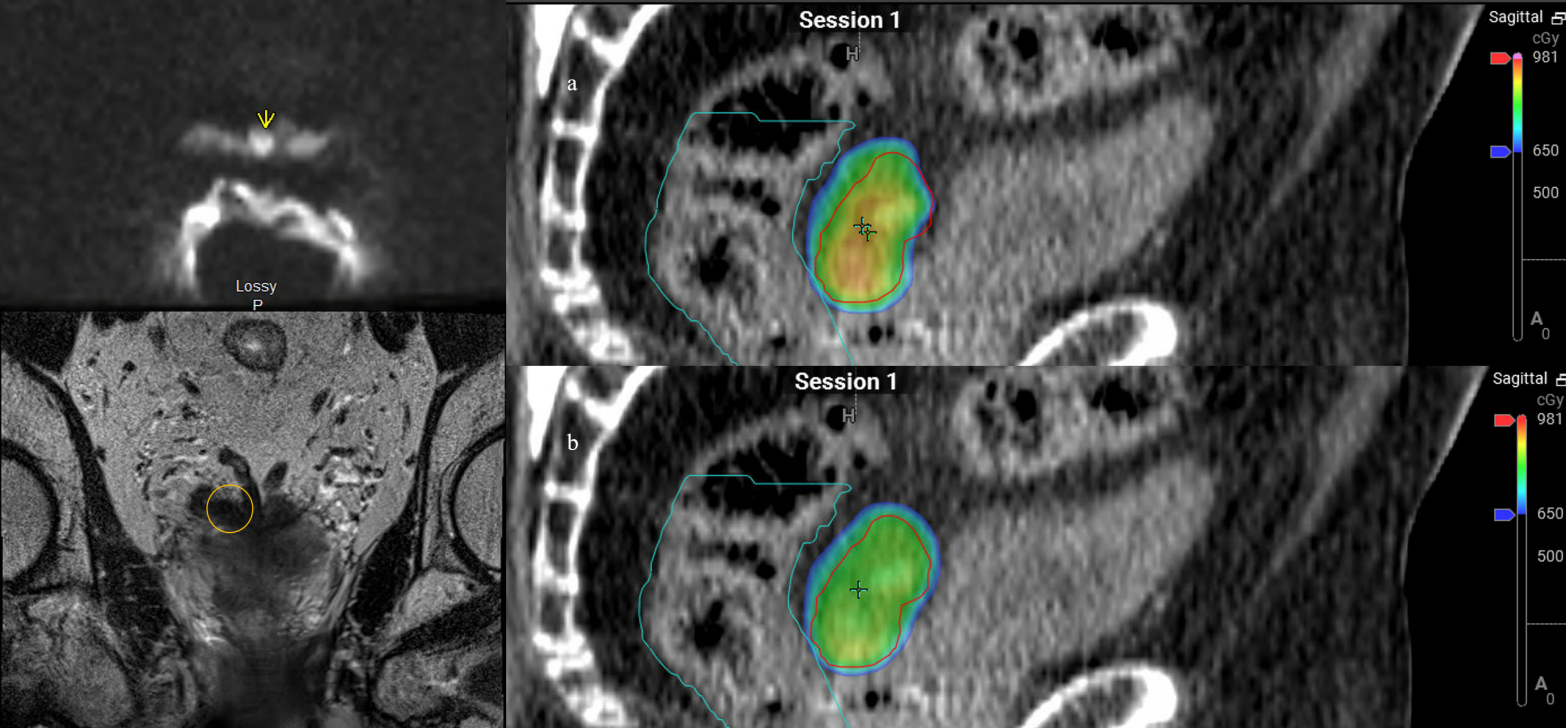
Patient 4. Left panel: diffusion weighted imaging with arrow indicating area of restricted diffusion and correlating location circled on coronal MRI. Right panel: Dose color wash of first fraction: **(A)** scheduled plan, **(B)** adapted (delivered) plan; red = PTV, cyan = rectum.

Patient 4 was diagnosed at the age of 61 with high-risk localized prostate cancer: T2cN0, a Gleason Score of 4 + 3 = 7, and an initial PSA of 51.02. He underwent pelvic and prostate radiation therapy with 45 Gy in 25 fractions to his pelvis, and 79.2 Gy in 44 fractions to his prostate and seminal vesicles along with 2 years of concurrent androgen deprivation with leuprolide. The patient’s PSA reached a nadir of 0.18, and his PSA began to slowly rise, causing him to meet the Phoenix criteria for failure at age 69. An F18-fluciclovine PET/CT revealed a focus of uptake in his seminal vesicle adjacent to the posterior wall of the bladder. MRI revealed a diffusion-restricted lesion in the right vas deferens at the junction of the seminal vesicle corresponding to the site of uptake on PET/CT. He underwent salvage treatment with once weekly SAbR ART (Ethos, Varian), 40 Gy delivered in five fractions of 8 Gy to the seminal vesicles. Adaptation was used in two of the five fractions to prioritize rectal dose, with a decrease in rectal D50% by 5%–7% and Dmax by 4%–5%. V40Gy coverage was more than 91% of the PTV for each of the delivered plans and congruent with the initial planning of 92% V40Gy. **Figure 4** demonstrates a representative sagittal comparison of the scheduled and adapted plan, with the improved dose homogeneity visually apparent in addition to the coverage of the target anteriorly with a decreased posterior extent of the 650 cGy line. No acute toxicity was experienced during treatment or at the 2-month follow-up. He received no concurrent or adjuvant systemic therapy. He had a robust response in PSA that decreased to 1.02, 3 months post-SAbR.

## Patient 5

**Figure 5:**
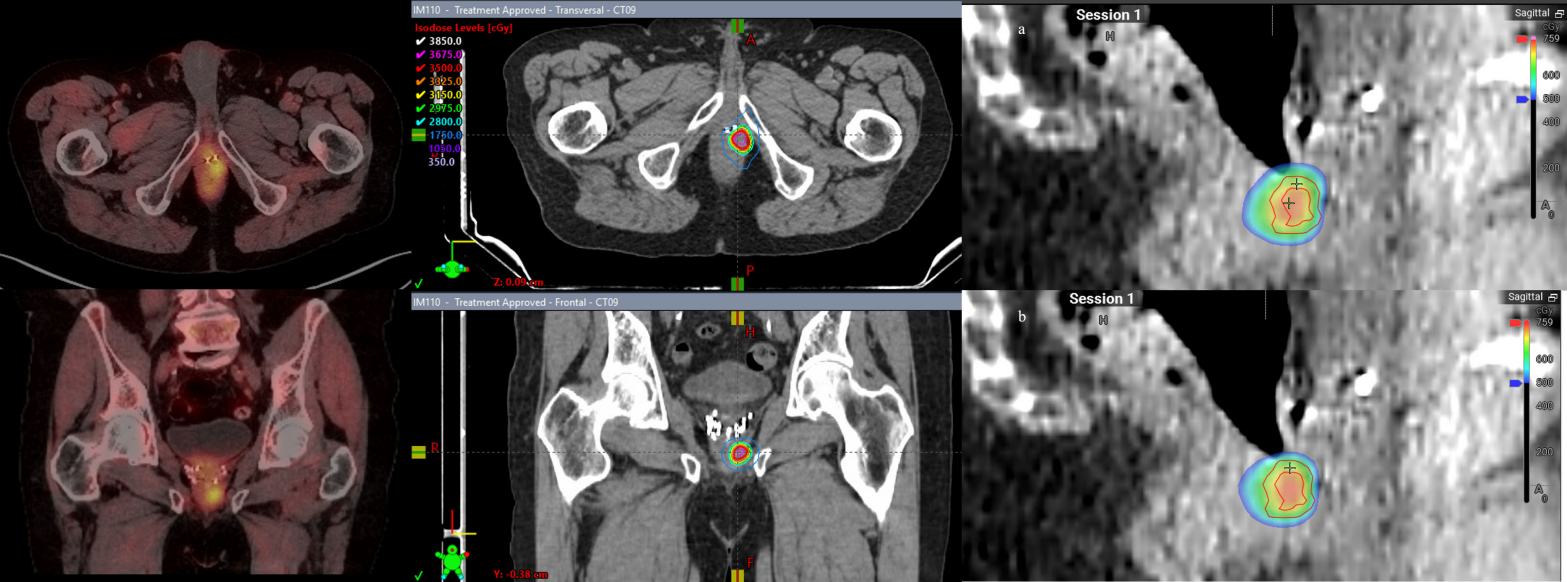
Patient 5. Left panel: Axial and coronal views of 18F-fluciclovine PET/CT with focal perirectal uptake inferior to implanted brachytherapy seeds. Middle panel: simulated ART plan with isodose lines. Right panel: Dose color wash of first fraction: **(A)** scheduled plan, **(B)** adapted (delivered) plan. Red lines denote the larger PTV 3,000 cGy and smaller PTV 3,500 cGy structures.

Patient 5 was diagnosed at the age of 58 with favorable intermediate risk prostate cancer: T1cN0, a Gleason Score of 3 + 4 = 7, and an initial PSA of 5.4. He underwent I-125 brachytherapy seed placement with initial improvement. He had a rising PSA but no identified focus and was started on long-term androgen deprivation with bicalutamide and dutasteride at which point he transferred care to our institution. His PSA nadir was 0.3 on ADT 10 years after initial RT and was generally stable on long-term bicalutamide, but started to have a slowly rising PSA over the next 6 years. At age 74, the patient’s PSA reached 3.0 and he underwent F18-fluciclovine PET/CT, revealing a focus of intense uptake in the left-sided root of the penis/perirectal space. MRI showed a 1.7-cm lesion along the left lower anterior rectal wall abutting the penile bulb, caudal to his I-125 seeds. A biopsy confirmed prostate cancer recurrence and a Gleason Score of 4 + 4 = 8. He received weekly SAbR ART treatment of 30 Gy in five fractions to the PTV with a SIB boost of 35 Gy to the GTV (**Figure 5**). Adaptation was used in all five fractions to improve coverage of the target. Thirty Gray PTV coverage was improved from approximately 96% to over 97%, while 35 Gy PTV was consistently improved from 80% to 90% to approximately 93% coverage, both approaching simulated V30Gy 99% and V35Gy 94% with rectal Dmax 3% lower with adaptation. This target was somewhat deformable due to bowel gas, thus lending well to adaptation. However, bowel gas caused significant artifacts on the cone beam CT; therefore, we used the implanted brachytherapy seeds as markers to help delineate caudal/cranial borders of the treatment field. The patient had rectal urgency after the third fraction during treatment, but this was resolved by the 2-month follow-up. He was started on abiraterone soon after starting SAbR. His PSA improved post-SAbR but was still detectable after treatment, prompting a repeat F18-fluciclovine PET 7 months post-SAbR, which showed an interval decrease in radiotracer uptake in the irradiated disease without another obvious site of disease.

## Discussion

This case series demonstrates the feasibility and application of ART and MRgRT in salvage re-irradiation of the prostate at our institution within the last year (**Table 1**). These rare cases illustrate a broad range of anatomical sites that carry a small risk of locoregional relapse after primary therapy for prostate cancer. Our series also differs from focal prostate salvage clinical scenarios where interstitial brachytherapy implants within the prostate gland are an accepted form of treatment (7). While our patients have not had an adequate follow-up to assess long-term toxicity or oncologic control, the acute toxicity profiles during treatment were mild or non-existent.

**Table 1:** Pertinent details of treatment course for this series of patients including treatment delivery time.

Patient	Primary therapy	Initial systemic therapy	Location of recurrence	Concurrent/adjuvant systemic therapy	SAbR dose	Total EQD2 (primary treatment + salvage)	Modality	Adaption	Duration of imaging, min (mean)	Duration of delivery, min (mean)	Total treatment time, min (mean)
**1**	Definitive RT, 79.2 Gy/44 fractions	Leuprolide	Perirectal	None	35 Gy in 5 fractions	159.7 Gy	MRgRT	No	3, 3, 3, 2, 2 (2.6)	9, 11, 13, 24, 9 (13.2)	62, 48, 42, 69, 53 (54.8)
**2**	RALP + salvage RT 5 years later	None	Prostatic fossa/posterior bladder wall	Leuprolide/darolutamide	35 Gy in 5 fractions	NA	MRgRT	No	3, 2, 3, 2, 3 (2.6)	13, 17, NA, NA, 7 (12.3)	48, 54, NA, NA, 42 (48)
**3**	Definitive RT, 79.2 Gy/44 fractions	None	Intraprostatic recurrence	Abiraterone/leuprolide	40 Gy in 5 fractions	183.2 Gy	MRgRT	No	7, 3, 3, 2, 5 (4)	23, 14, NA, NA, NA (18.5)	43, 72, NA, NA, NA (57.2)
**4**	Definitive RT, 79.2 Gy/44 fractions	Leuprolide	Seminal vesicle	None	40 Gy in 5 fractions	183.2 Gy	ART	Yes (2/5 fractions)	1, 1, 1, 1, NA (1)	10, 10, 10, 8, NA (9.5)	39, 40, 32, 35, NA (36.5)
**5**	I-125 LDR brachytherapy, 144 Gy	Bicalutamide	Perirectal	Leuprolide/abiraterone	30/35 Gy in 5 fractions	NA	ART	Yes (5/5 fractions)	NA, 2, 1, 2, 1 (1.5)	7, 10, 10, 6, 10 (8.6)	49, 43, 47, 40, 54 (46.6)

Equivalent dose in 2 Gy fraction (EQD2) calculations done with an alpha/beta of 1.5. NA, not applicable.

The major advantage of MRgRT in our case series was improved onboard imaging capabilities to safely delineate the targets from nearby soft tissue, ultimately providing the confidence needed to perform re-irradiation. While MRgRT is capable of ART, this was not necessary for patients 1, 2, and 3 as each target was precisely identified, organs at risk (OARs) were accurately reproduced on the day of the treatment, and the prescription dose was easily delivered. Therefore, a real-time decision on the day of the treatment was made to “adapt to position” rather than “adapt to shape.” MRgRT provided superior soft tissue delineation beyond what conventional CT or onboard CBCT imaging would provide. Furthermore, image acquisition continues during beam delivery, allowing for real-time intrafractional motion tracking without fiducial marker placement with MRgRT.

Adaptation to OARs or the deformable target was the major advantage of ART. In one patient, ART primarily improved high-dose boost coverage of the gross lesion while maintaining a safe rectal dose. In another patient case, ART decreased target coverage unacceptably due to over-prioritization of the OAR dose. The latter situation can be avoided at the pre-planning level by relaxing the OAR dose-limit constraints. Clinical judgement is required for safe and effective adaptive radiation delivery in these settings, and this begins with strong physician–planner communication to define “hard stop” constraints. At the time of delivery, the treating physician must decide whether to deliver the adapted plan, the scheduled plan, or restart the patient setup to attempt another adaptation.

A major challenge with ART and MRgRT has been the time required for treatment delivery. Pre-plans based on CT/MR simulations are overlaid with image acquisition on the day of treatment and must be adapted or translated to patient positioning and anatomic changes. Major contributors to prolonging treatment length are contouring time, plan optimization by the proprietary software, and per-fraction quality assurance, which are added time for treatment compared to standard linear accelerator-based treatments. In ART, this can add 10 to 15 min, while in MRgRT, it may add 30 to 45 min due to the need for image acquisition. Methods to reduce adaptation time include planning formulas [e.g., CTV = (prostate + SV) – (rectum + 1)] and avoiding over-designation of “influencer” structures, the structures that the planning software prioritizes sparingly with adaptive planning. Patients treated with full bladders are particularly challenging cases, as they may not tolerate the extra time required for plan optimization during ART/MRgRT. Contrastingly, rapid acquisition of images prior to delivery can show interval anatomic changes, such as bladder filling, adding yet another safety checkpoint prior to delivery. Patient setup can be modified to account for added treatment time, for example, having the bladder comfortably full instead of maximally full to allow for anatomic changes during adaptive planning so that OARs are optimally positioned at the time of repeat acquisition of images prior to delivery.

Our patients had variable anatomic locations for local recurrence of prostate cancer, with their cases highlighting the utility of both MRgRT and ART. Both cases allowed for safe treatment without the need for additional invasive procedures such as fiducial markers or biogel rectal spacers. Indeed, rectal spacers are of interest in the post-irradiated intact prostate setting with case series describing their success, as well as significant toxicity, possibly due to tissue fibrosis, impaired integrity, and unpredictable tissue plane dissection (8, 9). Based on the feasibility of our limited experience, ART should be evaluated as an alternative to brachytherapy in the salvage setting for radiation-recurrent prostate cancer. Indeed, focal implants are dependent on patient ability to undergo anesthesia and transperineal invasive procedures. Furthermore, acceptable access to the lesion of interest is also required. MRgRT/ART was chosen over salvage brachytherapy in these cases because of intolerance to anesthesia, patients’ preference for a non-operative therapy, prior prostatectomy, or locations of recurrence being deemed to be too challenging for an ultrasound-guided brachytherapy approach. Treatment in the re-irradiation setting will continue to improve with novel advances in radiation delivery and onboard imaging, though further studies are needed to validate new technology in these rare and challenging clinical scenarios.

## Data availability statement

The original contributions presented in the study are included in the article/supplementary material. Further inquiries can be directed to the corresponding author.

## Ethics statement

Written informed consent was obtained from the individual(s) for the publication of any potentially identifiable images or data included in this article.

## Author contributions

All authors contributed to the article and approved the submitted version.

## Acknowledgments

We thank Sepeadeh Rapdo for assistance in proofreading this manuscript.

## Conflict of interest

The authors declare that the research was conducted in the absence of any commercial or financial relationships that could be construed as a potential conflict of interest.

## Publisher’s note

All claims expressed in this article are solely those of the authors and do not necessarily represent those of their affiliated organizations, or those of the publisher, the editors and the reviewers. Any product that may be evaluated in this article, or claim that may be made by its manufacturer, is not guaranteed or endorsed by the publisher.
